# Identification of the Sixth Complement Component as Potential Key Genes in Hepatocellular Carcinoma via Bioinformatics Analysis

**DOI:** 10.1155/2020/7042124

**Published:** 2020-10-05

**Authors:** Di Mu, Feng Qin, Baoshan Li, Qian Zhou

**Affiliations:** ^1^Institute for Viral Hepatitis, Chongqing Medical University, Chongqing, China; ^2^Key Laboratory of Molecular Biology for Infectious Diseases, Ministry of Education, Chongqing, China; ^3^Department of Infectious Diseases, The Second Affiliated Hospital, Chongqing Medical University, Chongqing, China; ^4^Department of Laboratory Medicine, The Second Affiliated Hospital of Chongqing Medical University, Chongqing, China; ^5^Key Laboratory of Laboratory Medical Diagnostics, Chinese Ministry of Education, Chongqing Medical University, Chongqing, China; ^6^Department of Infectious Diseases, The People's Hospital of Shi Zhu, Chongqing, China; ^7^Department of Geriatrics, Chongqing Emergency Medical Center, Chongqing, China

## Abstract

The present study is designed to determine potential target genes involved in hepatocellular carcinoma (HCC) and provide possible underlying mechanisms of action. Several studies (GSE112790, GSE87630, and GSE56140) from the GEO database looking at molecular characteristics in HCC were screened and analyzed by GEO2R, which led to the identification of a total of 93 differentially expressed genes (DEGs). From the protein–protein interaction (PPI) network, we selected 13 key genes with high degree of variability in expression in HCC. Expression of three key genes (NQO1, CYP2C9, and C6) presented with poor overall survival (OS) in HCC patients by UALCAN. C6, which is a complement component, was found by ONCOMINE and TIMER to have low expression in many solid cancers including HCC. Besides, Kaplan-Meier plotter and UALCAN database analysis to access diseases prognosis suggested that low expression of C6 is significantly related to worse OS in LIHC patients, especially in advanced HCC patients. Finally, the TIMER analysis suggested that the C6 expression showed significant negative correlation with infiltrating levels of six immune cells. The somatic copy number alterations (SCNAs) of C6 were associated with CD4+ T cell infiltration in HCC. Taken together, these results together identified C6 as a potential key gene in the diagnosis and prognosis of HCC.

## 1. Introduction

Liver cancer has been ranked the fourth leading cause of tumor-related mortality worldwide, and about 841,000 new cases and 782,000 deaths worldwide are reported each year [1]. The World Health Organization estimated that over one million liver cancer patients will die in 2030 [2]. Approximately, 85%-90% of primary liver cancer has been reported to be hepatocellular carcinoma (HCC). Currently, the treatment of HCC involves multidisciplinary strategies including surgery, radiofrequency ablation, transarterial chemoembolization, and molecular targeting therapy [3, 4]. Despite recent advances in novel diagnostic and therapeutic technologies, the 5-year overall survival rate of HCC patients remains poor. Moreover, numerous gene networks and multiple signal transduction pathways are dysregulated in HCC. Thus, identifying potential predictive biomarkers and assessing possible underlying mechanisms of molecular dysregulation in HCC are vital for its prevention, diagnosis, and treatment.

Recently, several HCC clinical samples have been analyzed with high-throughput sequencing technologies and extensive bioinformatics. The rapid advancement of genome sequencing technology and microarray has facilitated our understanding of cancer genomics, allowing us to explore more effective therapeutic targets for HCC. As a result, precision medicine has been extensively explored in HCC and other tumors [5–7]. Although genome sequencing technology and microarray have helped achieve rapid progression of precision medicine, understanding the biological mechanisms of HCC has been limiting due to tumor heterogeneity reported by independent studies, most of which focus on a single cohort. Although HCC exhibits diverse genomic features, finding a commonality between heterogenous genomic profiles could be vital for our understanding of HCC's development and progression. Therefore, this study was designed to investigate the genes altered in common between three gene chip datasets in the context of HCC.

We chose three HCC gene chip datasets from Gene Expression Omnibus (GEO). The differentially expressed genes (DEGs) comparing nontumor and tumor tissue were assessed by GEO2R. We then utilized DAVID database for gene annotation and enrichment pathway analysis of DEGs. From the DEGs' PPI network, we identified key genes in common between the datasets. Overall survival (OS) associated with expression of the identified genes was assessed by UALCAN database. We found three key genes whose expression levels were related to poor survival in HCC patients. Literature retrieval results showed that expression of all genes identified, including NQO1, CYP2C9, and C6, except for the sixth complement component (C6), had been associated with poor HCC prognosis. In this study, C6 expression and its association with prognosis of various cancer including HCC was comprehensively investigated with the ONCOMINE, TIMER, Kaplan-Meier plotter, and UALCAN database. To further investigate the biological function of C6, we correlated its expression with tumor-infiltrating immune cells in HCC microenvironments by TIMER. These findings illustrated a vital role of C6 in HCC and provided a potential relationship between C6 expression and tumor-immune interactions.

## 2. Materials and Methods

### 2.1. Microarray Data and Identification of DEGs

We chose three gene expression profiles GSE112790 [8], GSE87630 [9], and GSE56140 [10] from GEO (http://www.ncbi.nlm.nih.gov/geo/) for analysis. All three datasets met our inclusion criteria: (a) the samples from *Homo sapiens* were divided into the HCC groups and the nontumor or adjacent groups; (b) more than ten samples were included in each dataset; and (c) the samples were assessed by mRNA expression profiling.

DEGs between the HCC groups and the nontumor groups in the three datasets were determined by the online tool GEO2R, using following cutoff criteria: adjusted *P* value should be less than 0.01, and the absolute value of log FC (fold change) should be above 1.5. Then, the overlapping DEGs from the three datasets were obtained and visualized by Venn 2.1.0.

### 2.2. The Enrichment Analysis of DEGs

Gene ontology analysis (GO), which provides information on the molecular functions (MF), biological processes (BP), and cellular components (CC), was utilized for annotation of DEGs. Besides, KEGG (Kyoto Encyclopedia of Genes and Genomes), which is an online bioinformatics resource, was used to perform the pathway analysis of DEGs. DEGs annotated with both GO and KEGG were analyzed and summarized with DAVID (the Database for Annotation, Visualization and Integrated Discovery). *P* < 0.01 was considered as the cutoff to explore the potential function.

### 2.3. Interaction of DEGs and Validation of Key Genes among DEGs

To identify PPI (the protein–protein interaction) among the DEGs and construct the DEGs' interactome, we used STRING (search tool or the retrieval of interacting Genes). The cutoff values were set as confidence score above 0.4. Furthermore, the Cytoscape software (version 3.6.0) was used that automatically integrates and presents the results from STRING. The CytoHubba app in Cytoscape may be helpful for analyzing DEGs.

### 2.4. Survival Analysis and Expression among Key Genes

UALCAN is an online database based on the transcriptome results from RNA-seq and clinical data in TCGA. It can, therefore, provide information on the relative expression of genes in tumor tissues compared with normal tissues. Furthermore, the database also provides information on the influence of gene expression level on patient survival with multiple clinicopathologic profiles [11]. In this study, we created box plots that displayed the relationship of the gene expression of key genes between HCC samples and normal samples, along with *P* value of survival obtained from Kaplan-Meier plotter. Furthermore, we also used another database GEPIA (Gene Expression Profiling Interactive Analysis, http://gepia.cancer-pku.cn/index.html) to validate the observed correlation of the key genes.

For further analysis, we chose one hub gene, namely C6, which had rarely been reported before in the context of HCC. First, the expression level of C6 across multiple cancers was obtained from the two independent databases in ONCOMINE which contained 715 cancer-related microarray datasets and in TIMER which based on TCGA database [12, 13]. Then, we remapped C6 into UALCAN database to investigate the expression and survival in specific cancer types. Search was limited to specific cancer types based on the result of the ONCOMINE and TIMER. The threshold was *P* value <0.01. Finally, the Kaplan-Meier plotter database including 10,461 cancer samples and survival data associated with 54,675 genes were screened for C6-associated survival in selected cancer types. *P* value less than 0.01 was regarded as statistically significant.

### 2.5. Analysis of Key Genes Expression and Immune Infiltration by TIMER

The online tool TIMER that includes 10,897 samples among diverse cancers in the TCGA database provides a comprehensive information on the relationship between gene expression and six different types of tumor-infiltrating immune cells including B cells, CD8+ T cells, CD4+ T cells, macrophages, neutrophils, and dendritic cells [13]. We investigated the relationship between expression of C6 and the abundance of immune infiltrates in HCC using the TIMER tool. We also correlated somatic copy number alterations (SCNAs) of C6 with immune cell enrichments in HCC using the TIMER tool. In this online tool, SCNAs of C6 in HCC were divided into four categories, such as diploid/normal, arm−level deletion, high amplification, and arm−level gain. Box plots indicated the distributions of every immune subset (B cells, CD8+ T cells, CD4+ T cells, macrophages, neutrophils, and dendritic cells) at each copy number status in HCC. The infiltration abundance in every SCNA category was compared to the diploid/normal by two-sided Wilcoxon rank sum test, and statistical significance was defined as *P* < 0.01.

### 2.6. Cell Culture

Four HCC cell lines were generously donated by the Institute for Viral Hepatitis, Chongqing Medical University. HCC cell lines were cultured in DMEM (Hyclone) medium supplemented with 1% penicillin and streptomycin and 10% fetal bovine serum in a 5% CO_2_ atmosphere at 37°C.

### 2.7. RT-qPCR Analysis

Total RNA from HCC cells was extracted with Trizol (Life technology, USA) following the manufacturer's protocol. RT-PCR Kit (TaKaRa, China) was used to amplify 2 *μ*g of total RNA. RT-PCR was used with the Bio-Rad. The RT-PCR reaction conditions were as follows: 95°C for 2 min; 40 cycles at 95°C for 30 s; Tm 52-60°C for 30 s and 72°C for 30 s, followed by 72°C for 2 min. GAPDH gene was selected as a reference gene. mRNA levels were assessed by the 2^-*ΔΔ*Ct^ method.

### 2.8. Statistical Analysis

All statistical analyses were performed with the SPSS 17.0 software, and continuous variables were described by means ± SD. *P* values < 0.01were considered statistically significant.

## 3. Result

### 3.1. Identification, Function Enrichment Analysis of DEGs, and Selection of Key Genes

**Figure 1:**
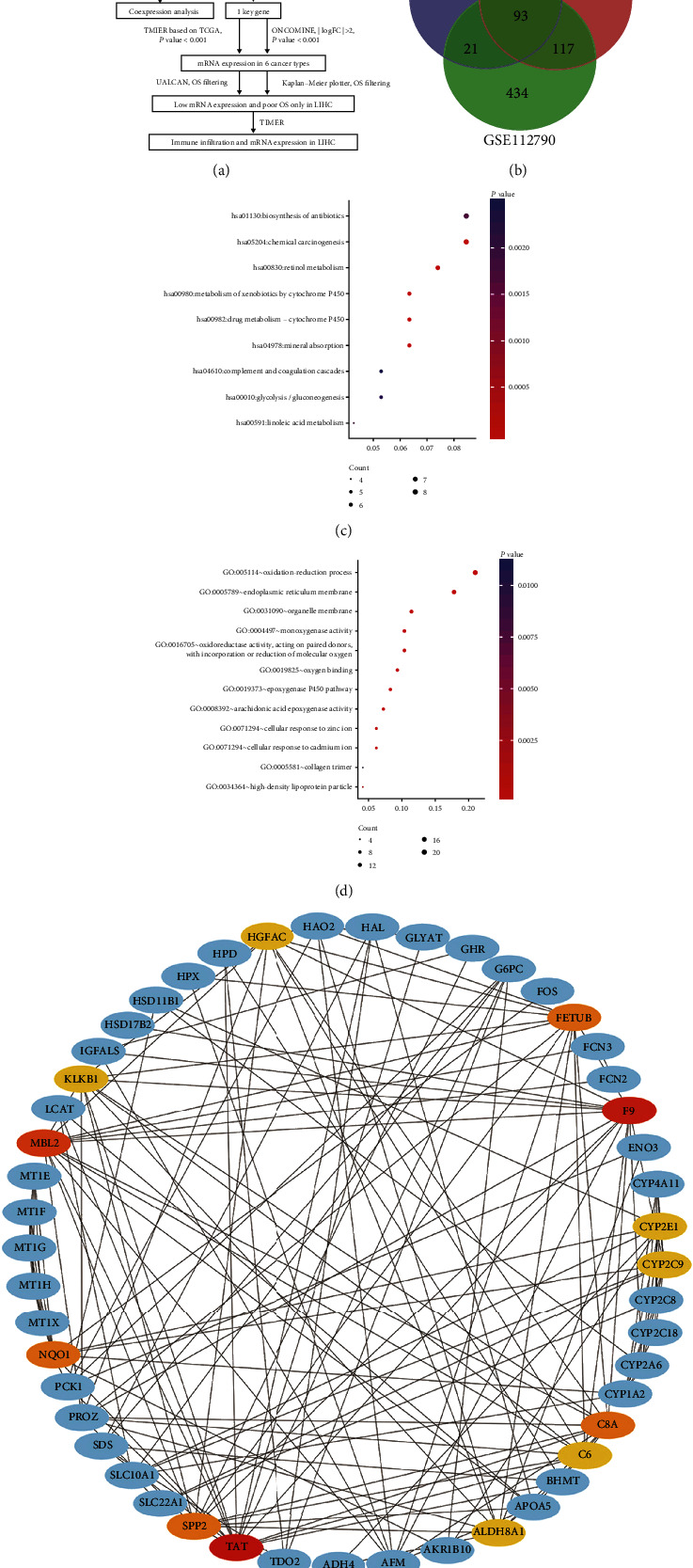
(a) The overall workflow of our work. (b) The data of Venn diagram about DEGs is extracted from the three GSE datasets (GSE112790, GSE87630, GSE56140). A total of 93 overlapping DEGs in the three datasets were obtained, as the following criteria: the absolute value of log FC > 1.5 and adjusted *P* value < 0.01. (c, d) GO enrichment and KEGG pathway analysis about DEGs were presented by scatterplot. Ratio variable was demonstrated in *x*-axis, and the terms of GO enrichment and KEGG pathway were shown in *y*-axis. The color and size indicated the range of *P* values and the gene number. (e) The analysis of DEGs interaction was performed by STRING and visualized with the Cytoscape software. The top 13 key genes with high degree were displayed with the first-stage nodes. Besides, the colors ranging from red, orange, to yellow represented the degree value is gradually decreased. The blue showed the first-stage nodes of other DEGs.

Figure 1(a) illustrates the overall workflow of our study for identification, validation, and functional analysis of DEGs. Based on the selection criteria, 3 HCC datasets were eligible for inclusion in this study.  Table 1 shows the details of each datasets that consists of GEO accession ID, study country, the number of samples, and platform information. There were 873 total DEGs in GSE112790 including 334 and 540 up- and downregulated genes, respectively. There were 403 total DEGs in GSE87630 including 82 and 321 up- and downregulated genes, respectively. Finally, there were 229 total DEGs in GSE56140 including 37 and 192 up- and downregulated genes, respectively. Together, we obtained a total of 93 overlapping DEGs from the three datasets (10 and 83 up- and downregulated genes, respectively), as illustrated in the Venn diagram (Figure 1(b) and Supplementary Table 1).

To further evaluate the biological function of DEGs, GO and KEGG enrichment analyses were performed using the DAVID database. With respect to KEGG pathway analysis, the most enriched pathways were chemical carcinogenesis and retinol metabolism pathways (Figure 1(c)). GO biological processes (BP) displayed epoxygenase P450 pathway, oxidation-reduction process, cellular response to cadmium ion, and cellular response to zinc ion pathways. For cell component (CC), the DEGs were enriched in organelle membrane, endoplasmic reticulum membrane, high-density lipoprotein particle, and collagen trimer. The molecular functions (MF) where the DEGs were enriched included oxidoreductase activity that acted on paired donors, with incorporation or reduction of molecular oxygen, monooxygenase activity, arachidonic acid epoxygenase activity, and oxygen binding (Figure 1(d)) .

Based on the information provided by STRING, which is a public database, we created the PPI network of DEGs by Cytoscape. Top 13 key genes with degree connectivity above 10 were selected by CytoHubba plug-in. The 13 genes were TAT, F9, MBL2, SPP2, FETUB, NQO1, C8A, HGFAC, KLKB1, ALDH8A1, CYP2E1, C6, and CYP2C9 (Figure 1(e)).

### 3.2. The mRNA Expression Level, Survival Analysis of Key Genes, and Experimental Validation of C6 Expression in HCC

**Figure 2:**
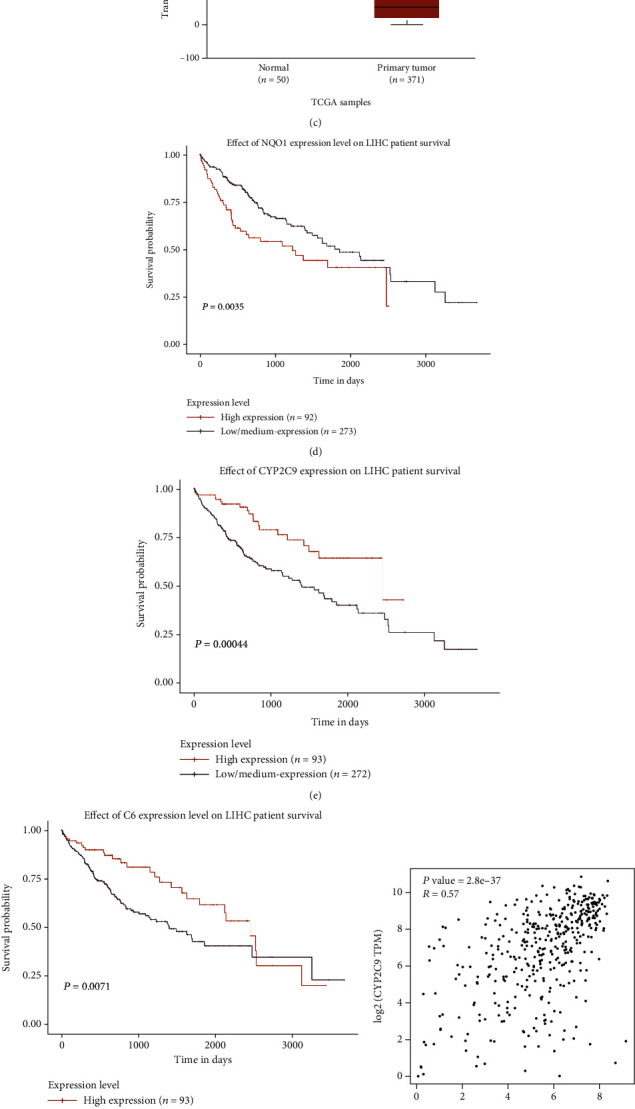
The relative expression of the NQO1 (a), CYP2C9 (b), and C6 (c) in HCC by UALCAN database. The relationship between the three key gene ((d) NQO1; (e) CYP2C9; (f) C6) expression and overall survival in HCC by UALCAN database. Color images are acquired according to the online UALCAN database. *P* < 0.01 was regarded as the significance. The correlated expression of C6 and NQO1 (g) and CYP2C9 (h) in HCC with GEPIA based on TCGA including 371 liver cancer tissue and 50 normal tissue. (i) C6 expression in four hepatoma cell lines and in control HL7702 liver cells. ^∗∗^*P* < 0.01, ^∗∗∗^*P* < 0.001.

We then investigated the association between the expression of the 13 key genes, and HCC prognosis from the available database UALCAN was investigated. Based on UALCAN, the mRNA expression of C6 (*P* < 1.00E10 − 12) (Figure 2(c)) was related to poor overall survival (OS) in HCC patients (Figures 2(c) and 2(f)). The expression of NQO1 (*P* = 1.62E10 − 12). (Figures 2(a) and 2(d)) and CYP2C9 (*P* = 5.00E10 − 15) (Figures 2(b) and 2(e)) was also associated with poor OS. Furthermore, we found that the expression of C6 and CYP2C9 was significantly lower in HCC than in normal tissues, while the NQO1 expression was statistically elevated in HCC patients. Among the three key genes, the C6 expression in HCC has been rarely reported before. Therefore, we assessed the correlations between the C6 expression and the two other key genes in HCC by GEPIA. GEPIA revealed that the C6 expression was significantly correlated with that of NQO1 (*R* = −0.31, *P* = 4.7e − 11) (Figure 2(g)) and CYP2C9 (*R* = 0.57, *P* = 2.8e − 37) (Figure 2(h)). In this study, the mRNA levels of C6 in four HCC cell lines were assessed. Compared to the normal human liver cell line, the C6 expression was also statistically downregulated in four human hepatoma cell lines (Figure 2(i)).

### 3.3. The C6 Expression Level and Prognostic Potential across Various Cancer

**Figure 3:**
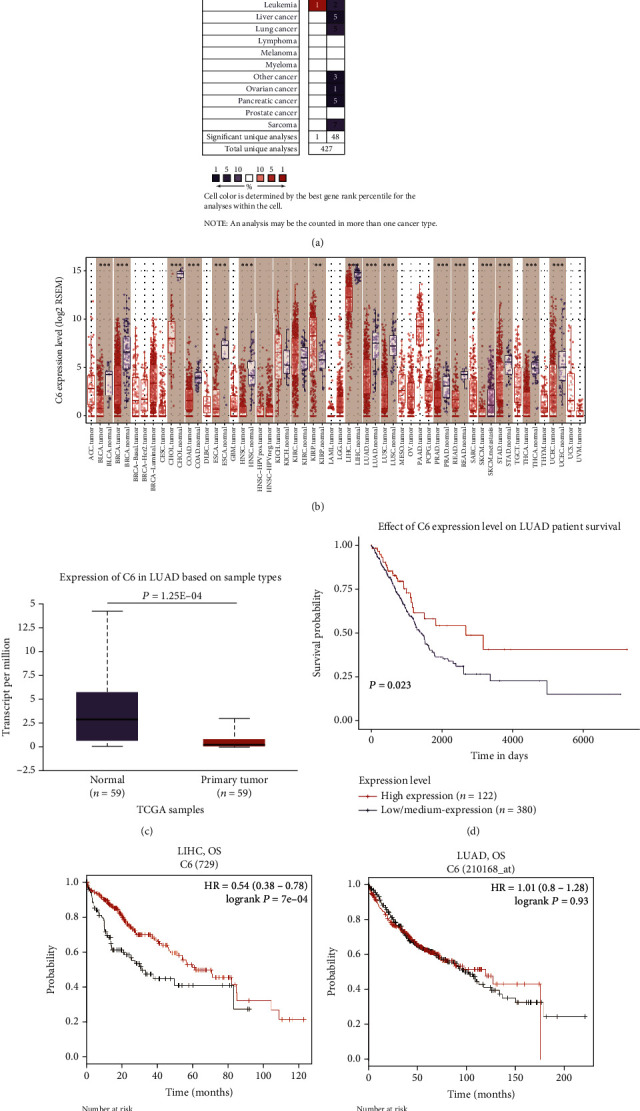
The expression of C6 across different cancer types was explored by the ONCOMINE (a) and TIMER (b) (^∗∗^*P* < 0.01, ^∗∗∗^*P* < 0.001) database. The relative expression (c) and overall survival (d) of C6 in LUAD by UALCAN database. Correlation between the C6 expression and the overall survival curves in LIHC (e) (*n* = 364) and LUAD (f) (*n* = 866) by Kaplan-Meier plotter databases. OS: overall survival; HR: hazard ratio.

**Figure 4:**
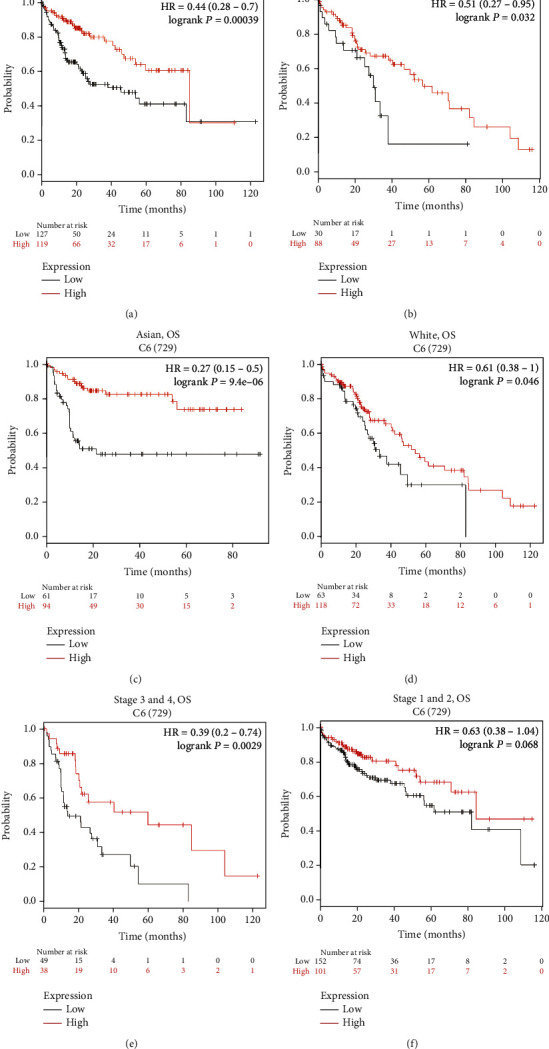
Overall survival curves in LIHC with association between the C6 expression and different clinicopathological feature by Kaplan-Meier plotter databases. The high and low C6 expression with different gender in male (a) (*n* = 250) and in female (b) (*n* = 121), with different race in Asian (c) (*n* = 158) and in White (d) (*n* = 184), with different stages in stages 3 and 4 (e) (*n* = 90) and in stages 1 and 2 (f) (*n* = 257). HR: hazard ratio.

To explore the difference of the C6 expression between the tumor and normal tissue, we analyzed C6 mRNA expression levels across various malignancies by ONCOMINE. Interestingly, we found that the C6 expression was significantly lower in most solid cancers except in leukemia, based on 1 dataset (Figure 4(a)). To further determine the C6 expression in human cancers, we examined RNA-seq results from various cancers in TIMER, based on TCGA database.  Figure 3(b) provides differences in the C6 expression in tumor and normal tissue in multiple TCGA malignancies. Fourteen cancer types showed extremely low C6 expression (∗∗∗*P* < 0.001), while the C6 expression was found to be higher in KIRC (kidney renal clear cell carcinoma) (∗∗*P* < 0.01). Since the C6 expression in SKCM (skin cutaneous melanoma) was compared with metastatic SKCM, this kind of tumor was considered unsuitable for further analysis in our study.

Based on the results in both ONCOMINE and TIMER databases, we found low C6 expression in BRCA (breast invasive carcinoma), HNSC (head and neck cancer), COAD (colon adenocarcinoma), LUAD (lung adenocarcinoma), LUSC (lung squamous cell carcinoma), and LIHC (liver hepatocellular carcinoma). We then used the UALCAN Database to investigate whether the C6 expression in these selected cancer types was correlated with diseases prognosis. The results demonstrated that the low C6 mRNA expression was correlated with relatively low OS in LUAD (*P* = 0.023) (Figures 3(c) and 3(d)), but the difference was statistically significant in LIHC (*P* = 0.0071) (Figure 2(f)). No significant differences were found between low C6 expression and OS. In addition, the lower C6 expression showed no influence on BRCA (*P* = 0.15), HNSC (*P* = 0.77), COAD (*P* = 0.9), and LUSC (*P* = 0.21). More details of the C6 expression in these four cancers are afforded in Table 2. Finally, we remapped C6 into the Kaplan-Meier plotter database to explore the impact of the C6 expression on survival rates in LUAD and LIHC. Poor prognosis in LIHC (HR = 0.55, logrank *P* = 0.0014) was observed to correlate with the lower C6 expression, while it showed no influence on LUAD (HR = 1.01, logrank *P* = 0.93). The findings validated the prognostic role of C6 in HCC patients.

### 3.4. Low C6 Expression Influents the Prognosis on Advanced HCC

To explore the potential mechanisms of the C6 expression level in HCC in more depth, we correlated the C6 expression with both disease prognosis and clinical features in Kaplan-Meier plotter databases. Low C6 expression was related to significantly poor OS in male patients (HR = 0.44, logrank *P* = 0.00039) (Figure 4(a)) compared to female patients (HR = 0.51, logrank *P* = 0.032) (Figure 4(b)). Furthermore, the low C6 expression had greater impact on the prognosis of survival in Asian HCC patients (HR = 0.27, logrank *P* = 9.4e − 06) (Figure 4(c)) compared to White HCC patients (HR = 0.61, logrank *P* = 0.046) (Figure 4(d)). Notably, the low C6 mRNA expression was associated with poor OS in stages 3 and 4 of HCC patients (HR = 0.37, logrank *P* = 0.00087) (Figure 4(e)) but was not correlated with OS of stages 1 and 2 HCC patients (HR = 0.73, logrank *P* = 0.27) (Figure 4(f)). The above data suggest that the C6 expression level is a great determinant impacting survival of advanced HCC patients.

### 3.5. C6's Expression Is Correlated with Immune Infiltration in HCC

**Figure 5:**
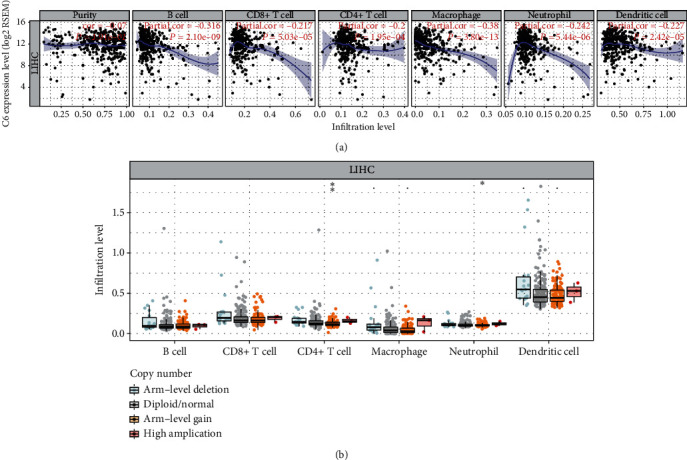
Correlation between the C6 mRNA expression and the abundance of immune infiltration level in LIHC (a). Each dot indicates a sample from TCGA dataset. Association of C6 SCNAs with immune infiltration in LIHC (b). The infiltration abundance in every SCNA category was compared to the diploid/normal. ^∗^*P* < 0.05 and ^∗∗^*P* < 0.01.

Cancer cells within the tumor microenvironment (TME) and neighboring tumor-associated noncancerous cells play important role in tumor biology. TIMER was used to investigate the potential associations of the C6 expression with tumor homogeneity and infiltrating immune cells. The results suggested that the C6 expression in HCC was not associated with tumor homogeneity (partial.cor = −0.07, *P* = 1.91e − 01) but was significantly negatively correlated with infiltrating levels of B cells (partial.cor = −0.316, *P* = 2.10e − 09), CD8+ T cells (partial.cor = −0.217, *P* = 5.03e − 05), CD4+ T cells (partial.cor = −0.2, *P* = 1.95e − 09), macrophages (partial.cor = −0.38, *P* = 3.80e − 13), neutrophils (partial.cor = −0.242, *P* = 5.44e − 06), and dendritic cells (partial.cor = −0.227, *P* = 2.42e − 05) (Figure 5(a)). Besides, the relationship between C6 SCNAs and infiltration of immune cells was also explored in TIMER. The results showed that the arm−level gain of C6 SCNAs in HCC was associated with CD4+ T cell (*P* < 0.01) and neutrophil (*P* < 0.05) infiltration (Figure 5(b)). Conversely, other C6 SCNAs such as arm−level deletion and high amplification showed no or weak relationship with infiltration of the above six immune cell types (Figure 5(b)).

## 4. Discussion

The process of hepatocarcinogenesis involves progressive accumulation of genetic alterations. The advent of high-throughput technologies has revolutionized cancer genomic research by focusing on genetic alterations and providing an effective approach to identify new biomarkers involved in HCC development and progression. Despite these advances, the molecular dysregulations leading to HCC remain unknown. In this study, we identified a total of 93 DEGs using GEO2R from the three GEO databases (GSE112790, GSE87630, and GSE56140). The GO pathways enriched with the DEGs included oxidoreductase activity, acting on paired donors, with incorporation or reduction of molecular oxygen, organelle membrane, and epoxygenase P450 pathway. Interestingly, these pathways were found to be mainly involved in chemical carcinogenesis and retinol metabolism. Among these DEGs, we chose 13 key genes. Using UALCAN, the expressions of these three key genes (NQO1, CYP2C9 and C6) were found to be associated with poor OS in HCC patients.

We further elucidated the progression of the three key genes in HCC. One of the three genes, NQO1 (NAD(P)H quinone dehydrogenase 1), is an antioxidant enzyme, which is known to be upregulated in HCC patients. A recent study has shown that elevated NQO1 could activate both the PI3K/Akt and MAPK/ERK pathways and promote metabolic adaptation in HCC [14]. Besides, the study demonstrated that an abnormally high expression of NQO1 would promote phosphorylation level of X-linked inhibitor of apoptosis protein (XIAP) and enhance stability of XIAP protein, thereby inducing tumor growth and inhibiting apoptosis in HCC [15]. Furthermore, NQO1 was identified as a predictive therapeutic marker of HCC by multiple other studies. Similarly, another gene CYP2C9 is a subfamily member of Cytochrome P2C (CYP2C), which has been found to participate in clinical drug metabolism. HCC chip assays have identified that the decrease of the CYP2C9 expression is involved in the progression of HCC [16]. Previous finding indicated that the CYP2C9 expression is regulated by microRNA hsa-miR-128-3p in HCC [17].

The third gene, C6, is a member of the complement system and is involved in innate immunity. Complete primary structure and function of C6 was elucidated in 1989. C6 is located on chromosome 5p13 and synthesized mainly in the liver in the form of glycoproteins [18, 19]. It has been shown to be involved in the membrane attack complex (MAC) that has important role in immunity. Thus, C6 combined with other complement molecules acts as powerful immune effectors for elimination of nonself cells. Several diseases in humans have been indicated due to C6 deficiency [20, 21]. To our knowledge, little research has been done on the expression and biological function of C6 in carcinoma, including in HCC [22]. Therefore, we decided to further explore the C6 expression levels in various cancers types in independent datasets in ONCOMINE and in TIMER.

Based on this analysis, the C6 expression between normal tissues and cancer was found to be similar in diversified solid cancers. According to the results of ONCOMINE database, the C6 expression presented a significant decline in many solid cancers compared with normal tissues, with only one study showing an opposite result: the high C6 expression found in leukemia patients. Compared with normal adjacent tissues, the TIMER based on TCGA further indicated that the C6 expression was lower in 14 cancer types including HCC but higher only in KIRC. Moreover, the prognostic impact of the low C6 expression in LUAD and LIHC by UALCAN was consistent, demonstrating a reduced C6 expression associated with shorter overall survival (OS) in HCC and LUAD. Nevertheless, analysis of data from Kaplan-Meier plotter database suggested that low level of the C6 expression was strongly related to worse prognosis only in LIHC. In addition, the low C6 expression was found to be related with worse OS of HCC patients in stages 3 and 4, OS of male patients, and those in Asian. It is interesting to find the gender distinction between the C6 expression and OS in HCC patients. Previous findings have postulated that sex-based distinction in the immune system could be associated with the natural course of chronic inflammatory including cancer. Cancer mortality rates were higher in male than in female from the vast majority of cancers [23, 24]. Moreover, accumulating evidence illustrates men have weaker innate and adaptive immune responses than women [25, 26], so it is worthy for further exploring the potential molecular mechanisms of the gender distinction between the C6 expression and the OS in HCC patients. Besides, our results also suggested that C6 was decreased in four human cell lines compared with a control cell line via RT-qPCR analysis that provided some basis for further research on C6's function in HCC. These results imply that C6 is a strongly prognostic target in HCC.

A growing body of research has shown that the tumor-infiltrating immune cells influence the clinical outcomes and efficacy of chemotherapy and immunotherapy in HCC [27, 28]. In addition, previous studies indicated that the complement system could act as a liaison between innate and acquired immunity via regulation of the B lymphocyte function and modulation of T lymphocyte activity [29]. To date, the effects of complement system on the tumor microenvironment have been widely investigated in various cancer [30, 31]. However, the role of C6 in the TME has been inconclusive so far [32]. Thus, we investigated the relationship of the C6 expression in HCC with level of immune infiltration using TIMER. We found that the C6 expression level in HCC was not associated with tumor purity. Moreover, one of the important aspects that has emerged from this study is that there is strong negative relationship between the C6 expression level in HCC and the level of infiltration of six immune cell types (Figure 5(a)). Besides, our results demonstrated a moderate to strong decrease of neutrophil and cell CD4+ T cell enrichment in HCC with a particular C6 SCNA (Figure 5(b)). Therefore, the results demonstrated that the expression and genomic alterations of C6 in HCC were tightly associated with the extent of immune infiltration. However, further study is needed to explore the possible mechanisms by which the C6 expression and genomic alterations affect immune infiltration.

## 5. Conclusions

In summary, the decreased C6 expression in HCC is associated with worse prognosis and elevated immune cell infiltration. This finding suggests that C6 is likely to participate in immune cell infiltration and may be a potential biomarker for HCC diagnosis and prognosis.

## Figures and Tables

**Table 1:** Characteristics of the three datasets.

Dataset ID	Country	Number of samples	Platform
GSE112790	Japan	183T 15N	GPL570
GSE87630	Korea	64T 30N	GPL6947
GSE56140	USA	35T 34N	GPL18461

GSE: Gene Expression Omnibus Series; GPL: Gene Expression Omnibus Platform; T: tumor samples; N: normal samples.

**Table 2:** The correlation of the C6 mRNA expression and overall survival in other four cancer types with UALCAN database.

Cancer types	C6 mRNA expression		Overall survival
	Expression level^∗^	*P* value	*P* value
BRCA	Downregulation	*1.50E-05*	0.15
COAD	Downregulation	1.55E-02	0.9
HNSC	Downregulation	*1.66E-03*	0.77
LUSC	Downregulation	*6.41E-07*	0.21

BRCA: breast invasive carcinoma; HNSC: head and neck cancer; COAD: colon adenocarcinoma; LUAD: lung adenocarcinoma.^∗^Tumor tissue compared with normal tissue. Italic values represented *P* < 0.05.

## Data Availability

The open available data sources or tools are as following: (1) http://www.ncbi.nlm.nih.gov/geo/, (2) http://bioinformatics.psb.ugent.be/webtools/Venn/, (3) https://david.ncifcrf.gov/, (4) http://gepia.cancer-pku.cn/index.html, (5) https://www.oncomine.org/resource/login.html, (6) https://cistrome.shinyapps.io/timer/, and (7) http://ualcan.path.uab.edu/.

## Supplementary Materials

Supplementary materials Supplementary Table 1: compared with normal tissues, a total of 93 DEGs were obtained from three profile datasets, including 10 upregulated and 83 downregulated genes in HCC tissue.
