# Supplementary material for: Identification of the Sixth Complement Component as Potential Key Genes in Hepatocellular Carcinoma via Bioinformatics Analysis
Source: Biomed Res Int. 2020 Oct 5;2020:7042124. doi: 10.1155/2020/7042124 (PMC7556077; doi:10.1155/2020/7042124)
Supplement: Supplementary materials — Supplementary Table 1: compared with normal tissues, a total of 93 DEGs were obtained from three profile datasets, including 10 upregulated and 83 downregulated genes in HCC tissue. [file 7042124.f1.pdf]

Supplementary:

Table 1:

Compared with normal tissues, a total of 93 DEGs were obtained from three profile datasets, including 10 upregulated and 83 downregulated genes in HCC tissue.

DEGs	Gene names
**Upregulated genes**	LCN2, SPINK1, TKT, CAP2, CDC20, TOP2A, AKR1B10, SQLE, UBE2C, NQO1.
**Downregulated genes**	ENO3, MT1G, LECT2, CYP2A6, SDS, ABCA8, SOCS2, CYP2C8, SLC22A1, HSD17B2, CYP39A1, SPP2, HAO2, HPD, MT1F, SLC25A47, FETUB, MFSD2A, PROZ, C8A, APOA5, MBL2, SLC10A1, LY6E, CYP1A2, MT1E, FCN3, GBA3, PDGFRA, ANXA10, CLEC4G, FXYD1, HPX, PCK1, GHR, CLEC1B, TAT, FOSB, CYP2C9, G6PC, BHMT, MARCO, CYP2A7, CYP2E1, LCAT, MT1H, LYVE1, CYP2C18, TDO2, HSD11B1, FOS, SHBG, PLAC8, HAMP, DNASE1L3, DCN, ALDH8A1, RND3, RDH16, CYP8B1, CXCL12, AFM, CRHBP, CIDEB, F9, CYP4A11, STAB2, HGFAC, IGFALS, MT1X, HAL, FBP1, ADH4, MT1M, GLYAT, CETP, GLS2, EGR1, C6, APOF, SRPX, FCN2, KLKB1.

DEGs differentially expressed genes.
